# Draft Genome Sequence of the Virulent *Clostridium chauvoei* Reference Strain JF4335

**DOI:** 10.1128/genomeA.00593-13

**Published:** 2013-08-15

**Authors:** Laurent Falquet, Sandra P. Calderon-Copete, Joachim Frey

**Affiliations:** Biochemistry Unit, University of Fribourg and Swiss Institute of Bioinformatics, Fribourg, Switzerlanda; Vital-IT, Swiss Institute of Bioinformatics, Lausanne, Switzerlandb; Institute for Veterinary Bacteriology, University of Bern, Bern, Switzerlandc

## Abstract

*Clostridium chauvoei* is the etiological agent of blackleg, a disease of cattle and sheep with high mortality rates, causing severe economic losses in livestock production. Here, we report the draft genome sequence of the virulent *C. chauvoei* strain JF4335 (2.8 Mbp and 28% G+C content) and the annotation of the genome.

## GENOME ANNOUNCEMENT

Blackleg, which is caused by *Clostridium chauvoei*, is a classical cattle epidemic disease. Similar to those of *Bacillus anthracis*, spores of *C. chauvoei*, which are released by diseased or dead cattle, survive in the environment for decades and can infect animals grazing on contaminated pastures (“champs maudits”). Hence, cattle must be vaccinated against blackleg in areas where *C. chauvoei* is endemic. The molecular evolution of *C. chauvoei* is expected to be slow due to the extended time period in which the bacterium remains in a dormant state (i.e., in the form of a spore).

The objective of this project was to establish a genomic reference sequence of a virulent strain of *C. chauvoei* isolated in Switzerland in 2004 that was investigated in detail for its toxin and sialidase biosynthesis properties (1, 2). For this purpose, genomic DNA sequencing of strain JF4335 was carried out at the Genome Technology Facility of the University of Lausanne on Pacific Biosciences (PacBio) and Illumina GAIIx machines using standard protocols. The genomic DNA was purified according to previous publications (1), and two libraries of 2 kbp and 5 kbp were prepared. The resulting data were quality controlled with FastQC (http://www.bioinformatics.babraham.ac.uk/projects/fastqc/) and assembled on the Vital-IT platform (http://www.vital-it.ch) using the Allora assembly module and the P_ErrorCorrection module of the SMRT pipeline 1.3.3 provided by the manufacturer. The combination of the two libraries of different sizes allowed us to correct for the PacBio’s huge error rate for large sequences (approximately 15% indels). The draft genome is composed of 12 contigs covering a genome size of 2,825,126 bp, with an N_50_ of 2,181,430 bp. In addition, we identified a potential plasmid of 5,566 bp.

The draft genome sequence was annotated using a pipeline we developed (3) and was deposited at the European Nucleotide Archive (ENA) (4).

### Nucleotide sequence accession numbers.

The sequences were deposited at EMBL under accession no. CBML010000001 to CBML010000011 for the genome and HG323816 for the plasmid.
